# Supplementary material for: Regulation of the JMJD3 (KDM6B) histone demethylase in glioblastoma stem cells by STAT3
Source: PLoS One. 2017 Apr 6;12(4):e0174775. doi: 10.1371/journal.pone.0174775 (PMC5383422; doi:10.1371/journal.pone.0174775)
Supplement: S1 Fig — (PDF) [file pone.0174775.s001.pdf]

Supplementary Figure 1

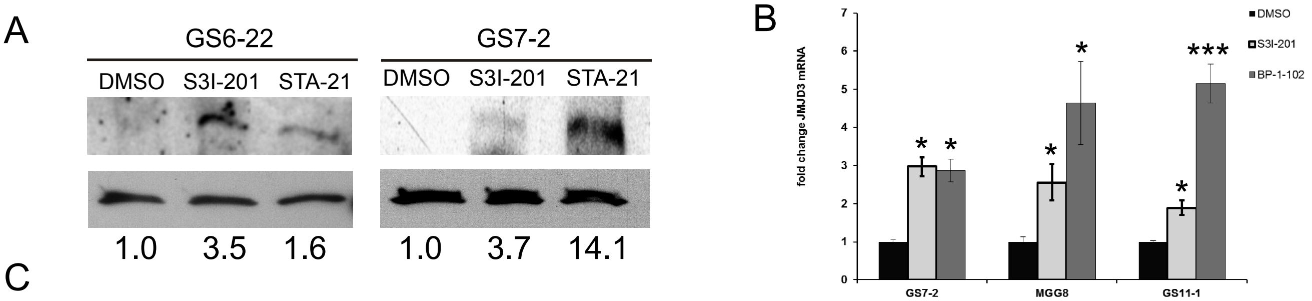

C

AAGGAAGAGCTGGGGCTAAAGGGAAGGTGCCCAAGAGAGGGGAGCGCTGTGCCAGTCGGGGGTCCAGTCGGCACCAAAGCGAAAGGGTG
GGGGGGGCGTGAAGGAGGAAGTGAAACGAGTTCTGGGTTCTTTAAGGGAGTGGTGGTGAGAGTGGGACAAGGGTGATGTCACTGCCTG
TCGGGCCCCGCCCTCCTCCCTCCCTCAGGACCCCCCCCCCCGCAGTACATTTACACACACCGCTTCCGCTGCGCAAGTAGCCACGTCAC
GGGCAACCCCCGAAATCCCCCCTCCCTTGGGGGGCGGGGGCCACGTGGGCCGTGGGGAACCGCGTGGCCCCGGCGGGGAAGGGCAGAGA
GGATACGCGAGCCACGAGAGAGGGGCCACTCGGGACCGCGGTGCGCGACTTCTCGGAAGTTGGGGGACTAAAAGGGGTCTGATTTTGGG
GGTCGGTCGGTATTGAAGACCCAGAAAGCCAG

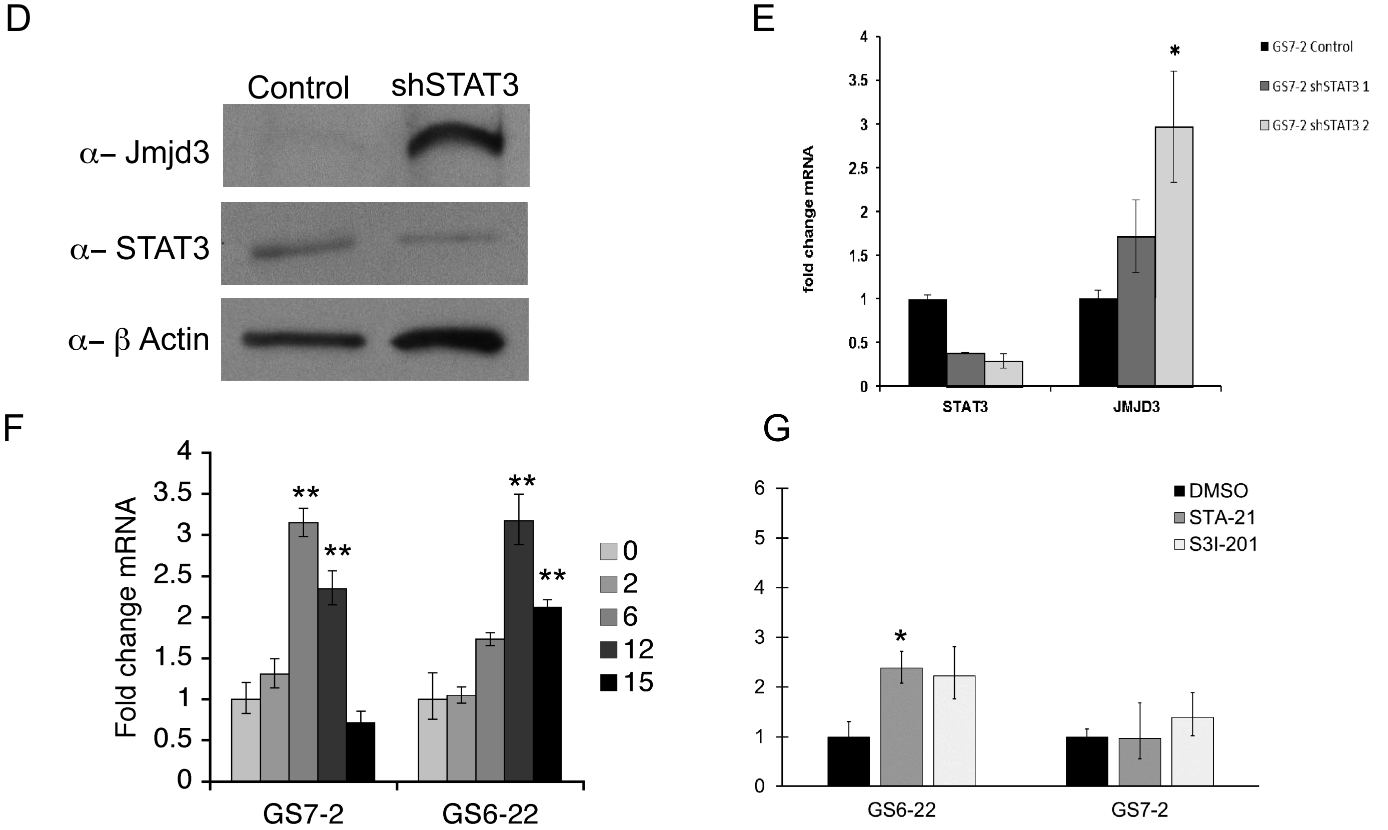

Supplementary Figure 1. STAT3 represses JMJD3 expression in glioblastoma stem cells. A. Treatment of GS6-22 or GS7-2 cells with the STAT3 inhibitor S3I-201 (50 uM) or STA-21 (50 uM) causes upregulation of JMJD3 protein levels. Cells were lysed for immunoblotting after 8 hours of drug treatment. JMJD3 protein levels relative to control were calculated using ImageJ and are displayed below each lane. B. RT-qPCR of GS7-2, MGG8 and GS11-1 cells treated with S3I-201 or BP-1-102 demonstrates that Jmjd3 mRNA is upregulated 6 hours after inhibitor treatment. The MGG8 line was obtained from Wakimoto et. al (2012). Values represent the fold change relative to control cells for three experiments. * $p < 0.05$ and *** $p < 0.001$ compared to DMSO control (Student's t test, two-tailed). C. Segment of the JMJD3 first intron (shown to be an alternate promoter in De Santa et al 2007) amplified in Figure 1D. Underlined letters signify the primer sequences, while bold underlined sequence represents the conserved STAT3 binding site (Chr17:7,747,573 UCSC GRCh37/hg19) which corresponds to the consensus binding site TTCNNNGAA (Horvath et al., 1995). In the murine JMJD3 promoter this sequence is TTCCCAGAA. D. Knockdown of STAT3 using an shRNA-containing lentivirus leads to the upregulation of Jmjd3 protein in GS7-2 cells, four days after selection in puromycin. E. Knockdown of STAT3 also leads to Jmjd3 mRNA upregulation. Values represent the fold change relative to DMSO treated cells for three experiments; bars SD (*$p<0.05$). F. GS6-22 and GS7-2 cells were differentiated for 15 days, and RNA was isolated at the timepoints indicated. Values represent the fold change of JMJD3 mRNA relative to control cells for biological triplicate. Bars represent variation, calculated as described in materials and methods. ** $p <0.01$; * $p < 0.05$ compared to DMSO control (Student's t test, two-tailed). Unlabeled bars are not significant. G. GS6-22 and GS7-2 cells were treated with S3I201 (50uM) or STA-21 (50 uM) for 24 hours, and then subjected to qPCR using primers to UTX. Values represent the fold change relative to controls for three experiments. Bars represent standard deviations, which were calculated as described in materials and methods. * $p <0.05$ compared to DMSO control (Student's t test, two-tailed). Unlabeled bars are not significant.
