# Supplementary material for: Regulation of the JMJD3 (KDM6B) histone demethylase in glioblastoma stem cells by STAT3
Source: PLoS One. 2017 Apr 6;12(4):e0174775. doi: 10.1371/journal.pone.0174775 (PMC5383422; doi:10.1371/journal.pone.0174775)
Supplement: S2 Fig — (PDF) [file pone.0174775.s002.pdf]

Supplementary Figure 2

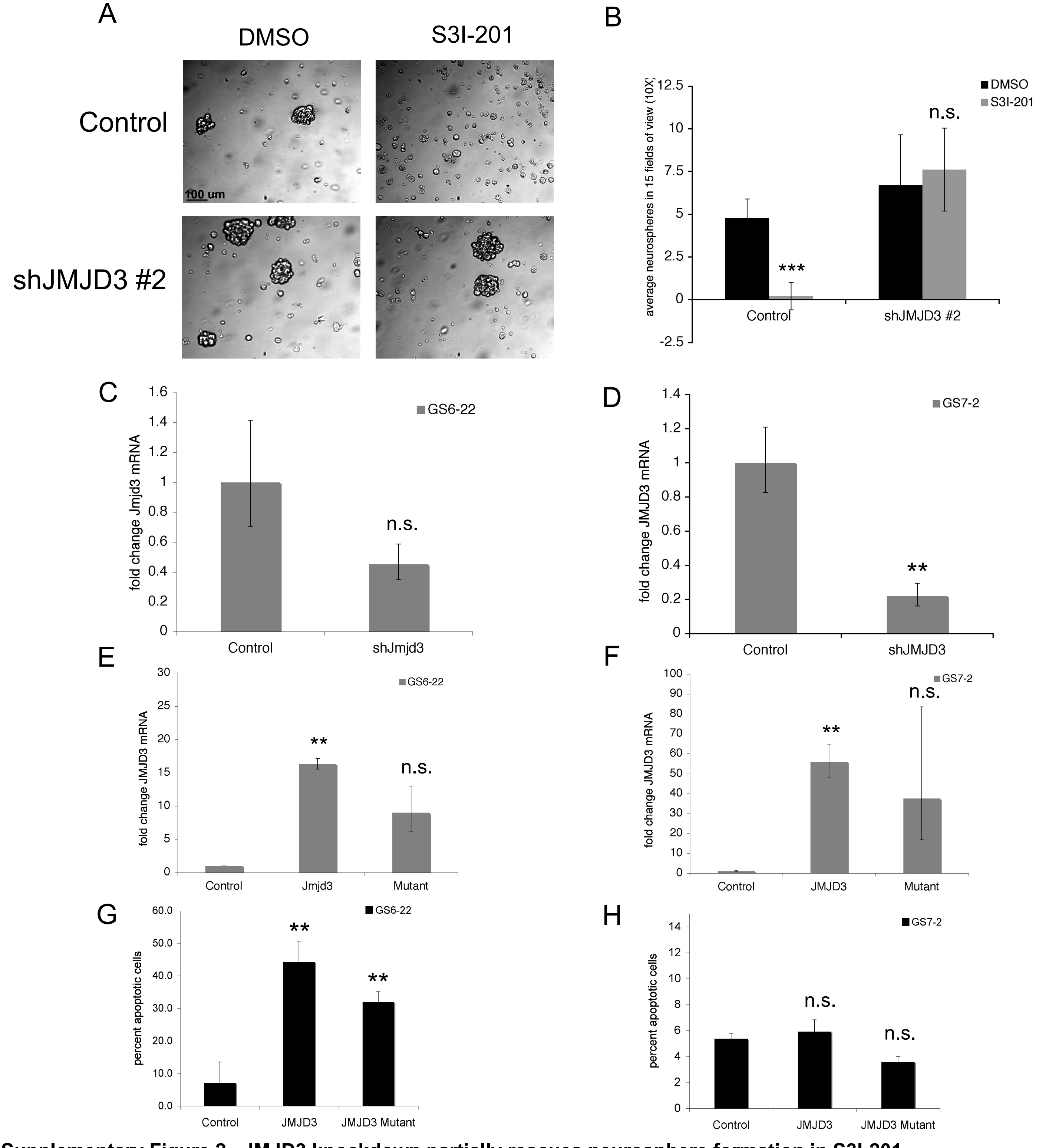

Supplementary Figure 2. JMJD3 knockdown partially rescues neurosphere formation in S3I-201.
A. GS6-22 cells were infected with a second shRNA to JMJD3, and plated in either DMSO or S3I-201 (50 uM). Representative images were taken 6 days after inhibitor treatment. B. Quantification of neurosphere formation after 6 days of drug treatment. Data represents the average number of spheres per field for one experiment (15 total fields per condition). *** $p < 0.001$; (Student's t test, two-tailed, compared to DMSO). Knockdown of Jmjd3 mRNA was 45% compared to shLuc control (data not shown). C. GS6-22 cells infected with shRNA to JMJD3 (Figure 2A) had 55% knockdown of JMJD3 mRNA compared to controls as assessed by qRT-PCR ($p = 0.07$). D. GS7-2 cells infected with shRNA to JMJD3 had 78% knockdown compared to controls (Figure 2A). Values represent the fold change relative to control cells for triplicate experiments. Bars represent standard variation, calculated as described in materials and methods. n.s.indicates not significant ($p>0.05$); ** $p < 0.01$ (Student's t test, two-tailed). E. GS6-22 and GS7-2 (F) cells were lysed, RNA extracted, and subjected to RT-qPCR. n.s. indicates $p > 0.05$ ($p = 0.07$); ** $p < 0.01$ compared to control. (Student's t test, two-tailed). JMJD3 overexpression is significantly higher in the GS7-2 cells than in the GS6-22 cells ($p < 0.01$). G. and H. Percent apoptotic cells was assessed in GS6-22 (A) and GS7-2 (B) cells with BrdU and 7-AAD staining. Values represent the average percentage of sub G0/G1 cells in 3 experiments; bars represent SD of the mean. n.s. indicates not significant; $p > 0.05$. ** $p < 0.01$ (Student's t test, two-tailed; each compared to control infected cells).
