# Supplementary material for: Regulation of the JMJD3 (KDM6B) histone demethylase in glioblastoma stem cells by STAT3
Source: PLoS One. 2017 Apr 6;12(4):e0174775. doi: 10.1371/journal.pone.0174775 (PMC5383422; doi:10.1371/journal.pone.0174775)
Supplement: S3 Fig — (PDF) [file pone.0174775.s003.pdf]

Supplementary Figure 3

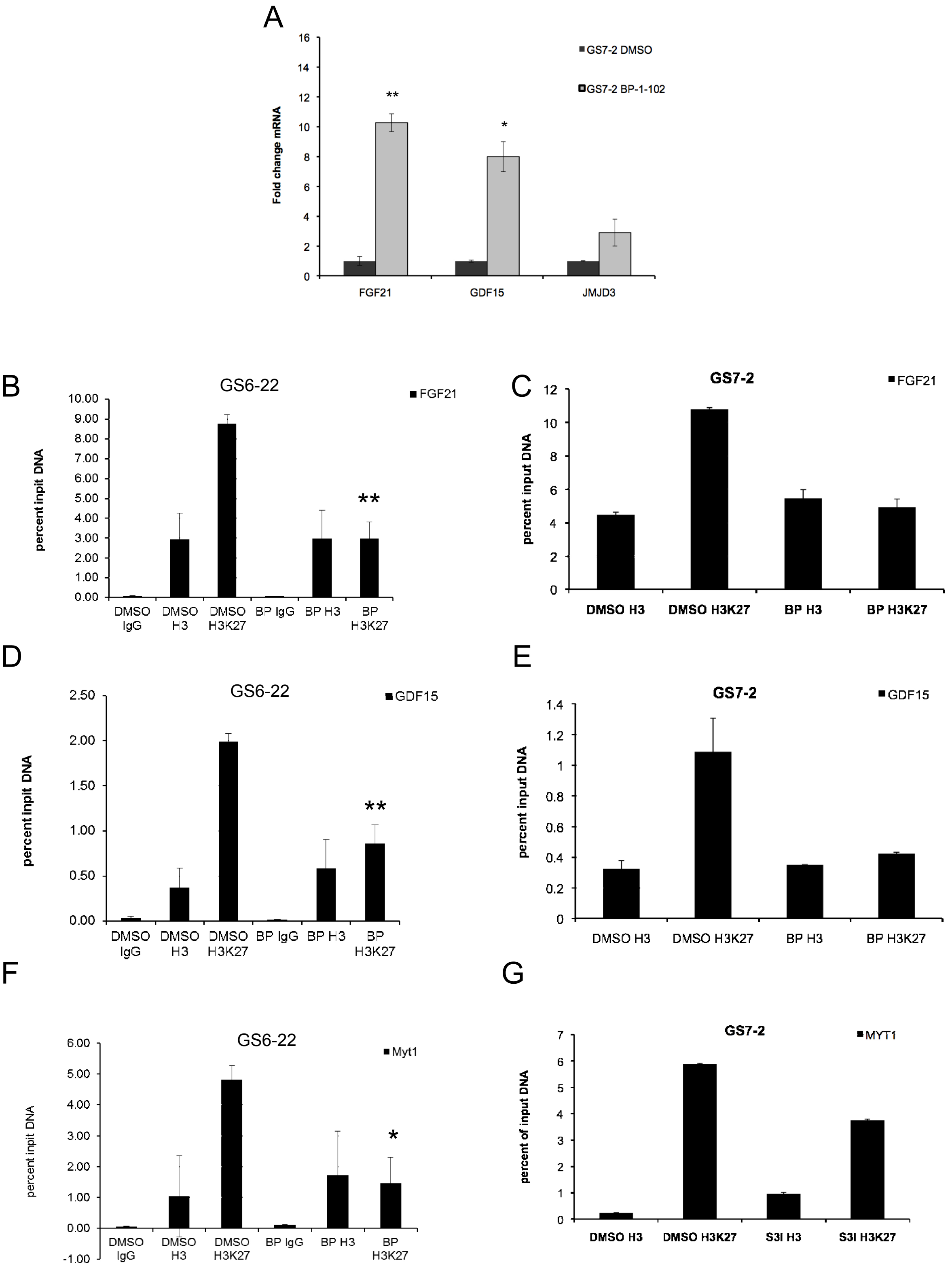

Supplementary Figure 3. Neural gene induction and H3K27 demethylation in BP-1-102-treated GBM-SC. A. RT-qPCR analysis of FGF21, GDF15 and JMJD3 expression in GS7-2 cells treated with BP-1-102 (15 uM) for 6 hours. Values represent the fold change relative to control cells; bars SD. B-G. GS6-22 and GS7-2 cells were treated with BP-1-102 (15 uM) or DMSO for 6 hours, fixed, lysed, and subjected to chromatin immunoprecipitation with an antibody to H3K27 trimethylation or total histone H3. Quantitative PCR using primers to FGF21 (B,C), GDF15 (D,E) or Myt1 (F,G) genes was performed after immunoprecipitation and DNA purification. Data is displayed as percentage of input DNA. * indicates p value less than or equal to 0.05, ** indicates p value less than or equal to 0.01 (Student’s T test, two tailed; compared to DMSO treated cells).
