# Supplementary material for: Regulation of the JMJD3 (KDM6B) histone demethylase in glioblastoma stem cells by STAT3
Source: PLoS One. 2017 Apr 6;12(4):e0174775. doi: 10.1371/journal.pone.0174775 (PMC5383422; doi:10.1371/journal.pone.0174775)
Supplement: S4 Fig — (PDF) [file pone.0174775.s004.pdf]

Supplementary Figure 4

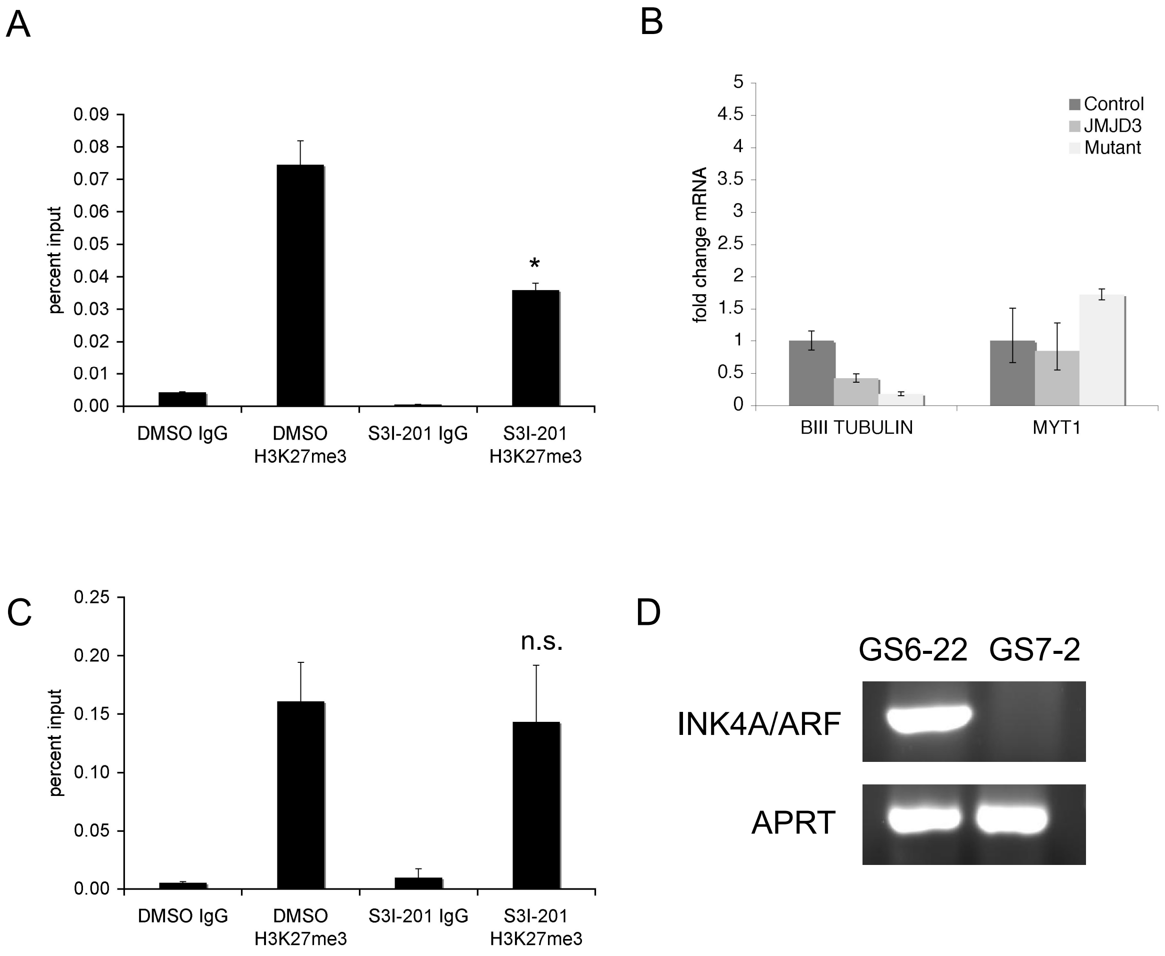

Supplementary Figure 4. *MYT1* and *INK4A/ARF* H3K27 methylation in GBM-SC. A. GS7-2 cells treated with the STAT3 inhibitor S3I-201 (100 uM) for 3 days were fixed, lysed, and subjected to chromatin immunoprecipitation with an antibody to H3K27 trimethylation. Quantitative PCR using primers to the *MYT1* promoter was performed after immunoprecipitation and DNA purification. Data is displayed as percentage of input DNA. Data represents 6 PCR reactions performed on two independent ChIP experiments. * $p < 0.05$ (Student's t test, two-tailed). B. GS7-2 cells infected with JMJD3, Mutant, or control retroviruses were subjected to qRT-PCR in order to assess marker expression. Data is analyzed as previously described. C. GS6-22 cells treated with the STAT3 inhibitor S3I-201 (100 uM) for 3 days were subjected to chromatin immunoprecipitation with an antibody to H3K27 trimethylation. Quantitative PCR using primers to a region of the Ink4A/Arf locus shown to be regulated by JMJD3 (Barradas et al., 2009) was performed after immunoprecipitation and DNA purification. Data is displayed as percentage of input DNA. Data represents 6 PCR reactions performed on two independent ChIP experiments. n.s indicates $p > 0.05$ (not signficant, Student's t test, two-tailed). D. GS6-22 cells possess an intact *INK4A/ARF* locus, but GS7-2 cells do not. GS6-22 and GS7-2 genomic DNA was subjected to PCR using primers to either the *INK4A/ARF* locus, or to the *APRT* (adenine phosphoribosyltransferase) gene as a control. Primers were constructed and PCR was performed according to the methods of Iwato et al (2000).
