# Supplementary material for: Regulation of the JMJD3 (KDM6B) histone demethylase in glioblastoma stem cells by STAT3
Source: PLoS One. 2017 Apr 6;12(4):e0174775. doi: 10.1371/journal.pone.0174775 (PMC5383422; doi:10.1371/journal.pone.0174775)
Supplement: S1 File — (PDF) [file pone.0174775.s008.pdf]

Supplemental Table Legends

Table S1. GS6-22 cells were treated with S3I-201 (50uM) or DMSO control for 3 days. ChIP Sequencing analysis using H3K27me3 antibody yielded a list of gene promoters that likely show a reduction in repressive H3K27me3 mark upon S3i treatment.

Table S2. GS6-22 cells were treated with S3I-201 (50uM) or DMSO control for 3 days. Microarray analysis yielded a list of genes that likely show 2 fold or higher gene expression upon S3i treatment.

Table S3. GS6-22 cells were treated with S3I-201 (50uM) or DMSO control for 3 days. Intersected list of genes showing both reduction in repressive H3K27me3 mark as well as 2 fold or higher gene expression upon S3i treatment.

Supplemental Experimental Procedures

qRT-PCR primers

Jmjd3 forward: CTACCCCCTTCACATGGCAG;

Jmjd3 reverse: CTCTGACTCGTACAGTTGCCC;

Myt1 forward: TGCTTGCCCCAAAGATTCAGA;

Myt1 reverse: AGTGCTCCTCACATAACTACTGG;

βIII-tubulin forward: GCCTCTTCTCACAAGTACGTG;

βIII-tubulin reverse: CCCCACTCTGACCAAAGATGAA;

β-actin forward: CCTGGGCATGGAGTCCTGTGG;

β-actin reverse: CTGTGTTGGCGTACAGGTCTT;

FGF21 forward: CTGTGGGTTTCTGTGCTGG;

FGF21 reverse: CCGGCTTCAAGGCTTTCAG;

GDF15 forward: ACCTGCACCTGCGTATCTCT;

GDF15 reverse: CGGACGAAGATTCTGCCAG.

Nano-ChIP-Seq Library preparation primers

Primer 1: GACATGTATCCGGATGTA **[X]** NNNNNNNNN**;**

where [X] denotes the custom barcodes for each sample as specified below:

ATCACG;

TTAGGC;

ACAGTG;

GATCAG;

TAGCTT;

GGCTAC;

Primer 2: GACATGTATCCGGATGT.

ChIP primers

Jmjd3 forward: AGGAAGAGCTGGGGCTAAAG;

Jmjd3 reverse: CTGGCTTTCTGGGTCTTCAA;

Myt1 forward: AGGCACCTTCTGTTGGCCGA;

Myt1 reverse: AGGCAGCTGCCTCCCGTACA;

FGF21 forward: CACAGTGCTGGGATTACCG;

FGF21 reverse: AGACGCTGGCCAACTAGAGA;

GDF15 forward: CAGGCACAGTGTCAACCAAG;

GDF15 reverse: AGGTTGCAGTGAGCCAAGAT.

Supplemental References

Wakimoto, H., Mohapatra, G., Kanai, R., Curry, W.T., Jr., Yip, S., Nitta, M., Patel, A.P., Barnard, Z.R., Stemmer-Rachamimov, A.O., Louis, D.N., et al. (2012). Maintenance of primary tumor phenotype and genotype in glioblastoma stem cells. Neuro Oncol 14, 132-144.
